# A Multiple Stakeholder Perspective for Evaluating Community-Based Dementia Care

**DOI:** 10.1093/geroni/igaa057.197

**Published:** 2020-12-16

**Authors:** Johan Suen

**Affiliations:** Duke-NUS Medical School, Singapore, Singapore

## Abstract

For holistic interventions and research on dementia, it is fundamental to understand care experiences from the perspectives of carers, care recipients, and care professionals. While research on care dyads and triads have highlighted the effects of communication and interactional aspects on care relationships, there is a lack of knowledge on how individual-contextual and relational factors shape the provision and receipt of care in terms of decision-making processes, resource allocation, and expectations of care outcomes. Thus, this paper sheds light on (i) how carers negotiate care provision with other important life domains such as employment, household/family roles and conflicts, as well as their own health problems, life goals, values, and aspirations for ageing; (ii) how older adults with dementia perceive support and those who provide it; (iii) the structural constraints faced by care professionals in delivering a team-based mode of dementia care; and, taken together, (iv) how community-based dementia care is impeded by barriers at the individual, relational, and institutional levels. Findings were derived from semi-structured interviews and observational data from fieldwork conducted with 20 persons with dementia (median age = 82), 20 of their carers (median age = 60), and 4 professional care providers. All respondents were clients and staff of a multidisciplinary and community-based dementia care system in Singapore. Our analysis indicates the impact of dementia care is strongly mediated by the interplay between institutional/familial contexts of care provision and the various ‘orientations’ to cognitive impairment and seeking support, which we characterised as ‘denial/acceptance’, ‘obligated’, ‘overprotective’, and ‘precariously vulnerable’.

